# The HPA – Immune Axis and the Immunomodulatory Actions of Glucocorticoids in the Brain

**DOI:** 10.3389/fimmu.2014.00136

**Published:** 2014-03-31

**Authors:** Marc-André Bellavance, Serge Rivest

**Affiliations:** ^1^Faculty of medicine, Department of Molecular Medicine, Neuroscience Laboratory, CHU de Québec Research Center, Laval University, Québec, QC, Canada

**Keywords:** HPA axis, glucocorticoids, glucocorticoid receptor, inflammation, microglia, stress

## Abstract

In response to physiological and psychogenic stressors, the hypothalamic–pituitary–adrenal (HPA) axis orchestrates the systemic release of glucocorticoids (GCs). By virtue of nearly ubiquitous expression of the GC receptor and the multifaceted metabolic, cardiovascular, cognitive, and immunologic functions of GCs, this system plays an essential role in the response to stress and restoration of an homeostatic state. GCs act on almost all types of immune cells and were long recognized to perform salient immunosuppressive and anti-inflammatory functions through various genomic and non-genomic mechanisms. These renowned effects of the steroid hormone have been exploited in the clinic for the past 70 years and synthetic GC derivatives are commonly used for the therapy of various allergic, autoimmune, inflammatory, and hematological disorders. The role of the HPA axis and GCs in restraining immune responses across the organism is however still debated in light of accumulating evidence suggesting that GCs can also have both permissive and stimulatory effects on the immune system under specific conditions. Such paradoxical actions of GCs are particularly evident in the brain, where substantial data support either a beneficial or detrimental role of the steroid hormone. In this review, we examine the roles of GCs on the innate immune system with a particular focus on the CNS compartment. We also dissect the numerous molecular mechanisms through which GCs exert their effects and discuss the various parameters influencing the paradoxical immunomodulatory functions of GCs in the brain.

## Activation Cascade and Regulation of the HPA Axis

Any imbalances to an organism homeostasis elicit a complex stress response that involves the coordinated activation of functionally overlapping neuroendocrine and autonomic systems. Among these critical systems is the hypothalamic–pituitary–adrenal (HPA) axis, which is triggered by stressors of various sources (physical, emotional, immunological, etc.) to provoke the systemic release of glucocorticoids (GCs).

**Figure 1:**
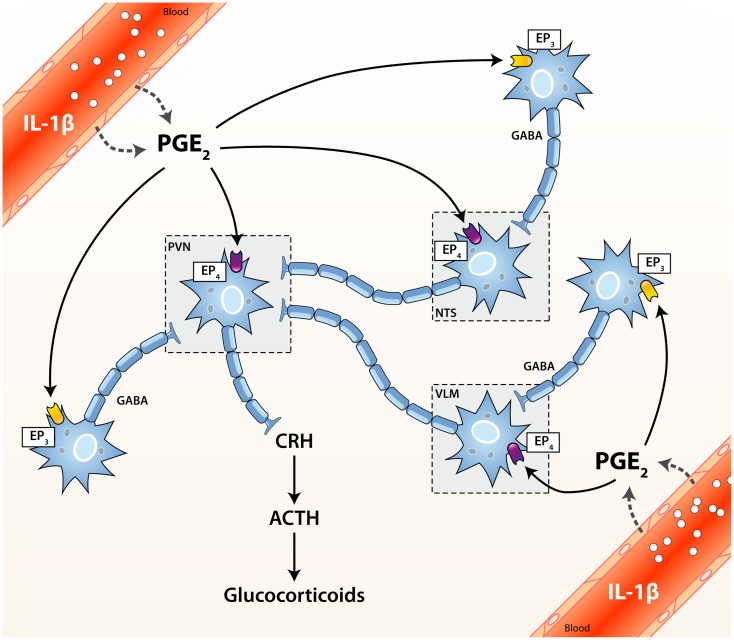
**Activation cascade of the hypothalamic–pituitary–adrenal (HPA) axis by systemic immune stimuli**. Integrated brain circuits trigger the parvocellular neurons of the PVN to release infundibular CRH, which stimulates the release of ACTH from corticotroph cells of the pituitary. ACTH reaches the bloodstream and finally induces the systemic release of GCs by the adrenals. PGE_2_ may activate or inhibit neurons through the EP_4_ and EP_3_ receptors, respectively. ACTH, adrenocorticotrophic hormone; CRH, corticotrophin releasing factor; EP_1–4_, PGE_2_ receptor subtypes; GABA, γ-aminobutyric acid (inhibitory); NTS, nucleus tractus solitarius (A2/C2 neurons); PGE_2_, prostaglandin of E2 type; PVN, paraventricular nucleus of the hypothalamus; VLM, ventrolateral medulla (A1/C1 neurons).

The activity of the HPA axis is regulated by multiple afferent sympathetic, parasympathetic, and limbic circuits (e.g., amygdala, hippocampus, and medial prefrontal cortex) innervating either directly or indirectly the paraventricular nucleus (PVN) of the hypothalamus. The PVN integrates converging stimulatory (catecholaminergic, glutamatergic, and serotonergic) or inhibitory (GABA-ergic) inputs, and thus represents a critical relay in the control of the HPA axis (1–3). The HPA axis is activated when secretory neurons of the medial parvocellular division of the PVN are stimulated, either directly or by relieving inhibitory inputs (Figure 1). As a result, corticotropin-releasing hormone (CRH) and arginine vasopressin (AVP) are both released in the portal circulation of the anterior pituitary gland. In turn, these neuropeptides trigger the secretion of adrenocorticotrophic hormone (ACTH) in the bloodstream by pituitary corticotrophs. ACTH then induces the production and the systemic release of GCs by the zona fasciculata of the adrenal cells (4–7).

Under basal conditions, the HPA axis exhibits a continuous oscillatory activity characterized by circadian and ultraradian variations. GCs are thus secreted in a highly pulsatile fashion throughout a 24 h cycle, displaying greater mean levels during the awake phase (8). The circadian rhythm of the HPA axis is orchestrated by the suprachiasmatic nucleus (SCN) of the hypothalamus (9) and the oscillating release of GCs is believed to optimize stress responses. Interestingly, the ultraradian pulsatility of the HPA axis was recently associated with pulses of glucocorticoid receptor (GR)-mediated transcriptional regulation (10). Upon stress, the intensity and duration of the HPA response both depend on the specific nature of the encountered stressor (5, 11, 12). The precise circadian or ultraradian phase at which stress occurs also profoundly influences the systemic release of GCs, since higher levels are secreted when the challenge coincides with rising pulses (13).

Multiple non-exclusive pathways participate in the activation of the HPA axis upon cerebral or peripheral immune challenges. When an immunogenic insult takes place in the brain, various inflammatory mediators produced locally may trigger the HPA axis. In contrast, multiple routes convey stimulatory signals from the periphery to the HPA axis when a challenge occurs outside the CNS. In this scenario, circulating immunogenic or inflammatory factors may access and activate regulatory neuronal circuits (either directly or not) projecting to the PVN via the fenestrated endothelium of the circumventricular organs (CVOs) or a disrupted blood–brain barrier (BBB) (14, 15). Alternatively, circulating immune ligands may also bind their cognate receptor(s) anchored in the luminal membrane of endothelial cells of brain capillaries. Hence, they can simultaneously engage numerous transduction signaling pathways that disseminate the activating cues to the HPA axis through the parenchymal release of diverse inflammatory messengers. To this end, distinct lines of evidence support a critical role of MyD88, COX-2, microsomal prostaglandin E synthase (mPGES-1), and prostaglandin E_2_ (PGE_2_) in relaying peripheral stimulatory signals to the HPA axis. Although the exact cell types at play are still actively debated, endothelial cells are widely acknowledged as pivotal in this activating cascade. The contribution of perivascular cells (PVCs) however remains a controversial topic since they were identified either as a negligible (16) or substantial source of PGE_2_ (17) in murine models of systemic inflammation. In addition to COX-2, more recent findings also support a role for COX-1 in activating the HPA axis (18, 19). Finally, afferent fibers of the vagus nerve may also signal peripheral inflammation to the brain and thereby activate the HPA response (20, 21).

The precise regulation of the activity of the HPA axis is of the utmost importance since both an excessive or insufficient release of GCs entail severe detrimental metabolic and immunological effects. As a matter of fact, chronic exposure to GCs results in various adverse side effects such as osteoporosis, diabetes, hypertension, dyslipidemia, and even neurodegeneration (5, 22). On the other hand, a deficient or blunted HPA axis is commonly observed in the clinic in a wide range of autoimmune and inflammatory diseases. Likewise, disrupting the HPA axis surgically (through adrenalectomy) or pharmacologically (with GR antagonists) compromises the survival of normally resistant mice to septic shock (23–27). The magnitude and duration of the HPA response is thus tightly controlled by autoregulatory feedback loops involving the adrenals, pituitary, PVN, and upstream corticolimbic structures such as the hippocampus, amygdala, and medial prefrontal cortex (28). As a result, the HPA response is terminated through the same neuronal circuitry that mediates its activation [reviewed in Ref. (2)].

## Actions of GCs on Immune Cells

The anti-inflammatory and immunosuppressive actions of GCs were first unraveled by the pioneering work of Kendall, Reichstein, and Hench more than 70 years ago and were since exploited in the clinic to treat a plethora of allergic, autoimmune, inflammatory, and hematological disorders as well as for preventing allograft rejection (29, 30). GCs exert immunomodulatory functions by acting on practically every immune cell type, by virtue of the nearly ubiquitous expression of the GR. The cell-specific actions of GCs, which underlie the long-recognized anti-inflammatory and immunosuppressive effects of this steroid hormone, are briefly highlighted below.

Glucocorticoids strongly influence the phenotype, survival, and functions of monocytes and macrophages. GCs have long been recognized to increase the phagocytic potential of these critical effectors cells and thereby stimulate the clearance of foreign antigens, pathogens, inflammatory cells, cellular debris, and other potentially harmful elements (31, 32). The steroid hormone also suppresses immunostimulatory functions and efficiently abrogates the production of pro-inflammatory mediators (such as cytokines, chemokines, and reactive oxygen or nitrous species) through various synergistic genomic and non-genomic mechanisms (33). In doing so, GCs promote an anti-inflammatory phenotype and expand the migratory activity and survival of these myeloid cells (34, 35).

Glucocorticoids perform similar key functions in dendritic cells (DCs). In addition to regulating the maturation, survival, and motility of these antigen-presenting cells, GCs also hamper their immunogenic functions. Indeed, the end product of the HPA axis restricts the capacity of DCs to stimulate T cells by preventing the up-regulation of various co-stimulatory molecules, such as MHCII, B7.2 (CD86), and CD40 (33, 36, 37). GCs can also convert DCs to tolerogenic cells, which promote the production of regulatory T cells (38, 39). This immunosuppressive effect critically relies on the GC-mediated expression of glucocorticoid-induce leucine zipper (GILZ), since this transcription factor appears as both necessary and sufficient for the induction of a tolerogenic state (40, 41). Recent work also indicates that the co-repressor DC-SCRIPT participate to this conversion (37). Interestingly, GCs exert distinct actions in immature and mature DCs and a disparate expression of various isoforms of the GR was recently found to underlie these divergent effects (42).

Another salient outcome of GCs administration is neutrophilia. The steroid hormone expands the number of circulating neutrophils by (1) increasing their egress from the bone-marrow to the bloodstream and by (2) concomitantly hindering their transmigration to inflammatory sites by alleviating the expression of cell adhesion molecules (43, 44). Paradoxically, GCs were also shown to promote or attenuate neutrophil apoptosis, respectively through Annexin A1 (45) or Mcl-1 and XIAP (46).

In contrast to neutrophils, GCs reduce the levels of circulating T cells by enhancing their migration back to the bone-marrow and secondary lymphoid organs (47, 48). GCs also trigger T cells apoptosis, notably through the up-regulation of BIM (49, 50). The relative expression of distinct GR isoforms is also believed to dictate the susceptibility of T cells to the pro-apoptotic effects of GCs (50). Additionally, the steroid hormone potently represses the production of pro-inflammatory cytokines, more specifically of those promoting Th1 and Th17 polarization (33). Accordingly, the targeted disruption of the GR in T cells (GR^lkc-Cre^ mice) produced hyperactive Th1 cells and increased mortality upon infection (51) while it precipitated the onset of disease in a murine model of multiple sclerosis (experimental autoimmune encephalomyelitis) (52). Complementarily, other lines of evidence suggest that GCs promote the differentiation of regulatory T cells (T_reg_), which are key suppressors of immune functions (53, 54). Finally, the steroid hormone may also interact directly with the signaling complex of the T cell receptor (TCR) after stimulation to inhibit its downstream transduction signaling pathways (55, 56). The actions of GCs in T cells thus heavily depend of the subtype targeted. Finally, GCs may also act on B cells and influence their survival, proliferation, and function (53, 57).

In addition to such prominent anti-inflammatory actions, a substantial body of knowledge now clearly indicates that GCs also have permissive and even stimulatory effects on immune processes. As a consequence, GCs are now more appropriately regarded as immunomodulators. The paradoxical actions of GCs are particularly evident in the CNS, where numerous lines of evidence either support a beneficial or detrimental role of the steroid hormone in various pathological contexts. In the next section, we aim to discuss the key functions assumed by the HPA axis and GCs in regulating the innate immune system of the brain in health and disease. We next dissect the multiple parameters defining the specific actions of GCs in these contexts.

## CNS Innate Immune System

The CNS was once regarded as an immune-privileged organ owing to the lack of lymphatic drainage, selective permeability of the BBB and apparent absence of immune responses. Significant advances in the last two decades have challenged this dogma and helped to considerably expand our understanding of the immune processes taking place in the CNS in health and disease. Robust inflammatory responses are elicited in neuropathological contexts such as infection, traumatic injury as well as autoimmune and neurodegenerative disorders by the resident innate immune cells of the brain, microglia. Dynamic research now aims at deciphering the crucial functions of microglia in preserving and restoring the brain homeostasis during immune challenges, injuries, and chronic diseases.

Microglia represent a heterogeneous cell population accounting for approximately 5–12% of total brain cells. They are distributed unevenly throughout the CNS, as grey matter and specific structures such as the hippocampus, basal ganglia, and substantia nigra typically all exhibit greater densities than the white matter (58–60). In resting conditions, microglial cells display a heavily ramified morphology and dynamically survey their immediate environment with highly motile but non-overlapping protrusions (61, 62). Upon encountering an endogenous or exogenous threat, microglia adopt an amoeboid morphology, show increased motility and promptly initiate an inflammatory response. In this context, activated microglia produce numerous inflammatory mediators and reactive species (oxygen or nitrous derived), which initially help to mobilize other immune cells and aim to restore homeostasis. Activated microglia also support neuronal survival and function by engulfing pathogens, cellular debris, or other neurotoxic entities and can synthesize various neurotrophic factors to promote tissue maintenance and repair (63, 64). However, the excessive or sustained activation of microglial cells (such as in chronic neurodegenerative diseases) usually produces significant inflammatory collateral damage and may hence fuel a vicious self-sustaining cycle driving further injury. Therefore, in order to preserve the fragile environment of the CNS many synergistic mechanisms are normally deployed to control microglia activity. Microglial cells represent prime targets of GCs in the CNS owing to a predominant expression of GR (65). Through these key innate immune cells, GCs therefore perform major regulatory functions on the innate immune system of the CNS in health and disease.

## GCs Bioavailability in the CNS

Owing that both endogenous and exogenous GC derivatives are highly lipophilic compounds, they easily diffuse through the BBB and act on nearly all cell types of the CNS. The bioavailability of endogenous and synthetic compounds can however diverge significantly according to three critical determinants: (1) the binding affinity to corticosteroid-binding globulin (CBG), (2) the uptake by various efflux pumps located at the BBB, and (3) the susceptibility to enzymatic metabolism. The endogenous bioactive form of GCs, namely cortisol in humans and corticosterone in rodents, is inactive while associated to plasma transport proteins such as serum albumin and CBG. CBG is a thermosensitive glycoprotein that binds up to 90% of circulating GCs in the bloodstream. The remaining 10% may also be associated to serum albumin. In consequence, the levels of free circulating GCs are usually about 5% (66, 67). Like most xenobiotics, synthetic GC analogs such as dexamethasone are efficiently expelled from the CNS by multidrug resistance (MDR) transporters expressed by the endothelial cells of the BBB (68). The efflux of free cortisol from the CNS is a major impediment and this phenomenon was suggested to underlie the preferential access of inactive GC metabolites to the brain.

Whereas plasma transport proteins and efflux pumps narrow the access of GCs to the brain, the activity of the steroid hormone is also regulated at the cellular level by the enzymatic interconversion of bioactive and inert GC species by 11β-hydroxysteroid dehydrogenases (11β-HSD). Briefly, 11β-HSD type 1 generates the bioactive form (cortisol or corticosterone) from inactive 11-keto derivatives while 11β-HSD type 2 catalyzes the opposite reaction. Interestingly, 11-keto derivatives do not bind to CBG in plasma nor are expelled by the efflux pumps of the BBB. As a result, 11-keto-GCs reach the CNS more readily than the bioactive form. 11β-HSD type 1 is widely expressed by both neurons and glial cells, whereas that of the type 2 isoform is more restricted (66, 68, 69). The distinct expression patterns of the two 11β-HSD enzymes in the CNS therefore also markedly influence GC signaling.

Collectively, these three pivotal parameters regulate GR signaling in the CNS by controlling the bioavailability of its ligands.

## Glucocorticoid Receptor

Glucocorticoids exert part of their biological effects by binding two proximate members of the nuclear receptor superfamily, namely the GR and the mineralocorticoid receptor (MR). Although GRs are ubiquitously expressed in the brain, they are most abundant in the PVN and the hippocampus. In contrast, MR expression appears mostly confined to a few limbic sites regulating salt appetite and autonomic outflow (70, 71). The binding affinity of cortisol is however 10-fold greater for the MR than the GR (66). MRs are thus heavily bound by basal/low levels of GCs while substantial ligation of GRs only occurs upon stress or when the highest ultraradian peaks are reached.

Like other nuclear receptors of its class, the GR is primarily composed of three characteristic domains: an N-terminal transactivation domain (NTD), a DNA-binding domain (DBD), and a C-terminal ligand-binding domain (LBD) (72). The human GR (hGR) is produced from a single gene (*NR3C1*, *chromosome 5q31.3*) and encompasses nine exons. The two best-characterized isoforms, namely GR-α and GR-β, arise from the alternative splicing of the exon 8 to distinct acceptor sites of exon 9. Alternative translation initiation sites further expand the variety of GR-α isoforms, producing height different proteins with a truncated NTD (GRα-A, GRα-B, GRα-C1, GRα-C2, GRα-C3, GRα-D1, GRα-D2, and GRα-D3). Finally, rearrangements in GR mRNA also yield three functionally distinct splice variants, namely GR-γ, GR-A, and GR-P (72, 73). The physiological significance of each GR isoform is beginning to emerge [elegantly reviewed in Ref. (74)], and remains best defined for the GR-α and β isoforms.

In the absence of ligand, GR-α is maintained in the cytoplasm but translocates to the nucleus upon ligation. In contrast, GR-β permanently resides in the nucleus where it selectively acts as a dominant negative inhibitor of GR-α (75). The relative abundance of the inhibitor GR-β, which normally represents only 1% of GR-α in the brain, is thus a key parameter in dictating GC responsiveness (76). Interestingly, pro-inflammatory cytokines were recently reported to increase the GR-β/GR-α ratio (77). Despite that endogenous or synthetic GC ligands do not bind to GR-β, previous work has nonetheless shown that its regulatory actions on transcription could be altered by the progesterone receptor (PR) as well as the GR antagonist mifepristone (RU486) (78). In the presence of other GR isoforms, GR-β appears to modulate (either positively or negatively) the transcription of a large set of genes that do not overlap with that of GR-α (77).

## Mechanisms of Transcriptional Regulation by the Glucocorticoid Receptor

The non-liganded GR-α is sequestered in the cytoplasm by a multiprotein complex that may be composed of heat-shock protein (HSP) 90, 70, 56, or 40, as well as co-chaperones p23, p60, Src kinase, and immunophilins FKBP51, 52 (22, 75, 79). These cytoplasmic chaperones conceal the nuclear localization signal (NLS) of the steroid receptor and thereby preclude its shuttling to the nucleus. Importantly, these chaperones also maintain GR-α in a conformation state that is optimal for ligand binding. The binding of a single GC molecule to a GR (1:1 ratio) provokes a conformational change that leads to the dissociation of chaperones and unmasks the NLS to importin proteins. The liganded GR is then transported to the nucleus where it profoundly modulates gene expression through multiple genomic and non-genomic mechanisms.

### Genomic mechanisms

**Figure 2:**
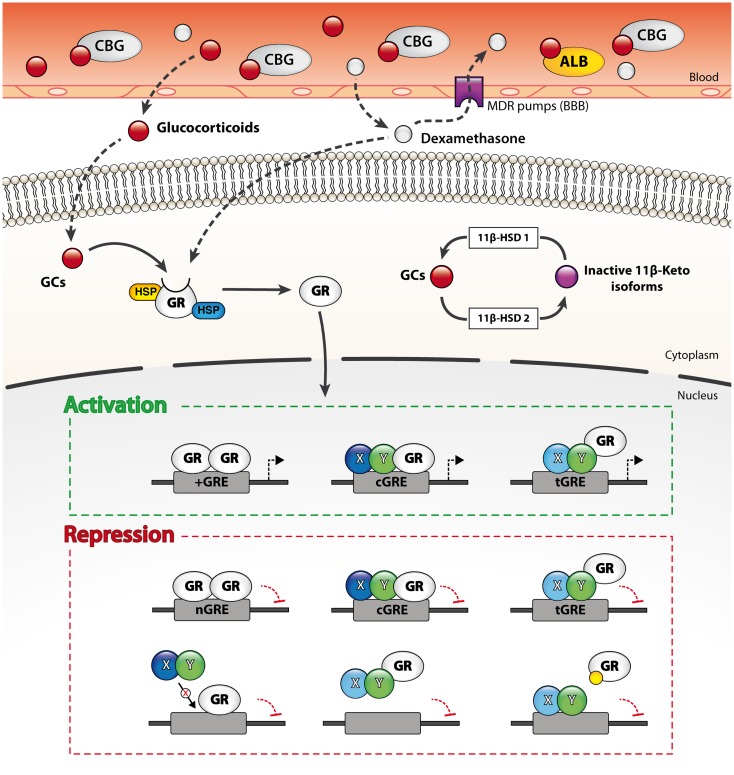
**Genomic and non-genomic mechanisms through which GCs regulate gene transcription**. Free circulating GCs easily diffuse through membranes such as the blood–brain barrier (BBB) and thus target both peripheral and CNS cells. The bioavailability of endogenous and exogenous GCs in the CNS is however limited at the organ level by efflux pumps expressed at the BBB and at the cellular level by enzymatic metabolism (11β-HSD enzymes). The unliganded GC receptor (GR) is sequestered in the cytoplasm by multiple chaperones. Ligation of the GR by a GC molecule (1:1 ratio) alters its conformation and results in the dissociation of the chaperones. The activated GR then translocates to the nucleus and dynamically modulate gene transcription through various mechanisms. Liganded GRs bind to four main types of GR-response elements (GREs). Activated GRs physically interact with DNA on simple (+GRE), negative (nGRE), and composite GREs (cGRE). The activated GR can also be recruited to other DNA-binding sequences (DBS) via protein–protein interactions (tGRE). Transactivation or transrepression activity of the GR is partly dictated by the type of GRE and its binding partners. Alternatively, GRs also regulate transcription through steric hindrance on DNA sites overlapping with GREs, by sequestering transcription factors from DNA and by competing for co-activators binding. Furthermore, liganded GRs may occupy other response elements by binding to overlapping GREs, sequester transcription factors from DNA and compete for co-activators.

Once in the nucleus, liganded GRs do not bind to DNA in a stable manner. They rather constantly shuttle between the nucleoplasm and GC-responsive elements (GRE) located in the promoter or enhancer regions of GC-responsive genes (80, 81). The variety of GRE is continuously expanding, but can nonetheless be divided into four broad categories: simple positive, composite, tethering, and the relatively novel simple negative GRE (nGRE) (Figure 2). Simple positive GREs (+GRE) represent imperfect palindromic sequences composed of inverted hexamers separated by a three base pair (bp) spacer. Each hexamer provides a binding interface for each monomer of GR homodimers. Simple GREs (+GRE) are deemed “positive” owing that they enable GR homodimers to stimulate gene expression (i.e., transactivation) through chromatin decondensation and recruitment of co-activators. GR-mediated transactivation typically induces the expression of potent signaling inhibitors (e.g., IκBα, MPK-1, IL-10, Annexin A1, GILZ, and SOCS proteins), which strongly interfere with salient immune signaling pathways such as those triggered by the Toll-like receptors (TLR) (82–85). Composite GREs (cGRE) represent chimeric sequences that are recognized by a GR monomer paired with another transcription factor (i.e., GR heterodimers). Tethering GREs (tGRE) stand out from other GRE types since they do not exhibit bindings motifs *per se* for the GR. They rather engage other transcription factor complexes that eventually recruit GRs through protein–protein interactions. As a result, tethered GRs do not physically interact with DNA at tGRE. Interestingly, both composite and tethering GREs enable direct transactivation or transrepression; the resulting effect on gene transcription is dictated by the transcription factors to which GRs are bound. For instance, tethering of a GR monomer was reported to increase the transcription of STAT3, STAT5, cAMP responsive element binding (CREB), and CCAAT/enhancer binding protein (C/EBP)-α responsive genes while inhibiting the activity of NF-κB, AP-1, activating transcriptions factors (ATFs), and IRF3 (72, 73, 86, 87). Additionally, the steric hindrance entailed by tethered GRs can interrupt gene transcription by hindering the recruitment and activity of the transcriptional machinery. In this line, very recent data point toward a role of GR:GRIP1 complexes in preventing the recruitment of PolII to initiation-controlled inflammatory genes such as IL-1α and IL-1β, and concomitantly promoting the accumulation of pause-inducing negative elongation factor, which precludes the release of PolII from the elongation block of genes like TNF-α, CCL2, and CCL3 (88). The fourth and last type is the simple nGRE. They are composed of two inverted repeats and are believed to be recognized by GR homodimers (89). In contrast to +GRE, GR activity at nGRE mediates the direct repression of transcription by recruiting the transrepressor nuclear receptor co-repressor 1 (NCoR) and silencing mediator of retinoid and thyroid hormone receptors (SMRT). Interestingly, nGRE were localized in many NF-κB and AP-1-responsive genes, which encode immune and inflammatory proteins. Finally, we note that through such genomic mechanisms, GCs regulate the expression and protein abundance of their own regulators (i.e., HES1) and that this feedback loop is mandatory for proper GC-mediated changes in gene expression (90).

### Non-genomic mechanisms

In addition to their genomic actions, liganded GRs also impact on gene transcription through various non-genomic mechanisms. These mechanisms do not require *de novo* protein synthesis and thus underlie the more rapid actions of GCs. Prominent examples include mRNA destabilization, competition for co-activators [e.g., CREB-binding protein (CBP), p300, and GRIP], and interference with the binding of transcription factors to DNA (91–94). Complementarily, freed cytoplasmic chaperones may also perform anti-inflammatory actions following their dissociation from the activated GR. For instance, the immunophilin FKBP52 was recently identified as a gene-specific regulator of GR functions (95). It should be noted that other types of GRs, such as those embedded in the cytoplasmic or mitochondrial membranes, were also proposed to regulate transcriptional activity but the mechanistic details underlying their origin and function remain ill-defined (96–98). Although definite proof is still lacking, it was posited that the membrane-bound GR might be a variant of the cytosolic form potentially arising from alternative promoters, differential splicing, or post-translational modifications (99). Of particular interest, ligation of GRs anchored in membrane lipid rafts by dexamethasone was recently found to abate intercellular communication through gap junctions in neural progenitor cells (100). Such mechanism might also operate in immune cells and influence their function by modulating cell–cell interactions and the immune synapse. Additional non-genomic actions of the liganded GRs may include direct interactions with membrane (e.g., GPCRs, ion channels, TCR), and cytoplasmic proteins (MAPK and phospholipases) that indirectly impact on transcriptional activity (76, 79, 101). For instance, dexamethasone was shown to potentiate CXCR4 signaling and even synergize with its ligand CXCL12 in resting T cells, presumably through the CD45- and GR-dependent activation of Lck and other downstream kinases (102). Therefore, GCs trigger various non-genomic and genomic signaling pathways that can act in concert to shape the function of immune cells. Finally, GCs may also entail non-specific and GR-independent effects by altering the physicochemical properties of plasma and mitochondrial membranes at high concentrations (101, 103) and by modifying the composition (104) or the formation (105) of lipid rafts in various immune cells.

### Functional insights from GR mutant mouse strains

Several lines of GR mutant transgenic mice provided key insights about the functions of the GR in regulating the HPA axis and immune processes (106). The GR^dim^ mouse, which carries a point mutation (A458T) preventing GR dimerization, is among the best characterized. Initial evidence suggested that classic transactivation by GRs was defective in these mice and that this mechanism was required for the full range of GC immunosuppressive actions (107, 108). This hypothesis is supported by prior work showing that repression of IL-1β, MIP-2, MCP-1, and IP-10 by GCs is hindered in macrophages isolated from GR^dim^ mice (109, 110). Contrastingly, other findings indicate that in GR^dim^ mice NF-κB and AP-1 target genes can still be silenced by GCs (107, 108), that the GC-inducible gene MKP-1 is normally expressed in macrophages (82) and that irritant dermatitis can successfully be treated with GCs (22). These contradictory data may arise from a residual transactivation activity of the GR in GR^dim^ mice since many other amino acids were found to enable the dimerization of the receptor (111). The single point mutation A458T engineered in GR^dim^ is thus unlikely to fully abrogate the formation of functional GR homodimers (30, 112, 113). Moreover, GR^dim^ regulatory defects were recently shown to be tissue, cytokine, and time-dependent, which further complicate the interpretation of the dimerization-dependent genomic actions of the GR (114).

The targeted knock-out of the GR was also achieved in a variety of cell populations or types to investigate the cell-specific functions of GCs. For instance, in the GR^lysMCre^ transgenic mice, GR expression is selectively reduced in macrophages, microglia, and other myeloid cells such as DCs, granulocytes, and mast cells. Upon MPTP intoxication (115) or intra-striatal injection of LPS (116), this strain exhibited greater neuronal loss, microglia reactivity, and expression of pro-inflammatory genes than age-matched controls. Of particular note, increased mortality was also observed in GR^lysMCre^ mice compared to controls following a peripheral injection of LPS (109). The GR^lysMCre^ mice therefore helped in uncovering the crucial actions of GR signaling in microglia and peripheral myeloid cells in the proper and timely regulation of the immune response in order to avoid secondary inflammatory damages. The role of GR signaling in endothelial cells was also recently tackled with two distinct transgenic lines. Using the Tie2-GRKO mice, one group recently revealed that GR activation in endothelial cells of brain vessels might be both necessary and sufficient to constrain the production of key structural proteins of the BBB following focal cerebral ischemia (117). Alternatively, the GR^EC KO^ mice displayed increased mortality, hemodynamic instability, and higher levels of inflammatory cytokines (TNF-α and IL-6) than controls during sepsis (118). Finally, the broad disruption of the GR allele in the CNS [GR^NesCre^ (5)] and its targeted deletion in the forebrain [forebrain-specific GR knock-out; FBGRKO mice] (119), or the PVN all resulted in the disinhibition of the HPA axis (120). However, current evidence indicates that GR disruption in the forebrain (FBGRKO vs. WT) did not influence neuronal death or damaged area in the hippocampus following an excitotoxic insult, nor the infarct volume in a model of focal cerebral ischemia (117). Elucidating the impact of the targeted GR deletion in specific cell populations on central immune processes as well as survival of neurons and glial cells in different pathological contexts will undoubtedly help to further decipher the complex context and cell-specific functions of GCs in the brain.

## Context-Dependent Actions of the Glucocorticoid Receptor

A myriad of GR, epigenetic, and contextual parameters underlie the cell-, signal-, and gene-specific actions of GCs. As exemplified above for GR-α and GR-β, GR isoforms have unique transcriptional regulatory profiles. GC actions on gene expression are thus first influenced by the relative abundance of the numerous GR isoforms in a given cell or tissue. In similar fashion, the differential expression of transcription factors, co-activators, co-repressors, and other binding partners are critical in tailoring the actions of GCs through the GR (22).

The activation state of a target cell and the specific transduction signaling pathways triggered at a given time further define the actions of the GR. Signaling pathways can fine-tune GR activity through various post-translational modifications including phosphorylation, ubiquitination, sumoylation, and acetylation (72, 121). Through their own effects on transcription, chromatin, and epigenome, cell signaling pathways further shape GR effects. For instance, TLR signaling pathways are subjected to suppressive actions of GCs in a signal- and gene-specific manner. To this end, Ogawa et al. found that the liganded GR sequesters the co-activator p65 from IRF3 and IRF7 homodimers on interferon-stimulated response elements (IRSE) on a specific set of genes following TLR4 and TLR9, but not TLR3 signaling (122). In such context, regulatory actions of GCs thus appear to be specified by MyD88 signaling. The liganded GR was also found to prevent the recruitment of IRF3 (which then acts as a co-activator) to p65 on NF-κB target genes upon TLR4 stimulation by LPS (94, 123). It was also reported that dexamethasone inhibit JNK upon TLR3 (TRIF) or TLR9 (MyD88) activation through the GR, but that the concomitant activation of both signaling pathways (or TLR4) yields to the resistance of JNK to deactivation by GCs (124). The same group recently reported similar results for SOCS1, a critical regulator of type I IFN transduction signaling pathways (83).

Lastly, genome wide analyses unraveled that changes as subtle as the permutation of a single bp in GREs modifies the conformation of the liganded GR. Through structure alteration, such changes orchestrate interactions with cofactors as well as the regulatory actions of activated GRs on transcription (125, 126). GRE sequences therefore represent key allosteric modulators. The ever increasing variety of *bona fide* GREs reported in the literature implies that the interactions of liganded GRs and DNA are much more flexible than previously anticipated.

## Paradoxical Actions of GCs on the Innate Immune System of the Brain

In addition to their long recognized and well characterized anti-inflammatory actions, accumulating evidence now indicate that GCs do not merely allow inflammatory responses to unfold but rather stimulate them under specific circumstances. Whether GCs play beneficial or detrimental roles in the CNS in health and diseases have been debated for decades, and the complex dichotomous and context-dependent actions of the steroid hormone certainly add to the confusion. The fact that GC actions evolve dynamically over time and the incomplete understanding of the parameters driving the CNS innate immune system toward tissue maintenance/repair or damage also warrant a careful interpretation of the literature. Here, we review experimental evidence supporting the bidirectional actions of GCs in the brain.

### Anti-inflammatory actions of GCs in the brain

Substantial evidence established that GCs restrain and/or terminate the innate immune response in the CNS following either a peripheral or cerebral challenge. Almost 20 years ago, exacerbated levels of pro-inflammatory cytokines were found in the brain of adrenalectomized mice following a sub-cutaneous injection of LPS (127–130). GCs were also shown to prevent the production of pro-inflammatory mediatory in cultured microglia primed with LPS (131, 132). Glezer et al. have reported concordant results *in vivo*, by showing that dexamethasone suppresses LPS-induced NF-κB activity in the murine brain (133). More recently, Munhoz and colleagues demonstrated that a severe or mild deficiency in plasma corticosterone significantly enhanced NF-κB activation in the brain of rodents challenged with systemic LPS (134). Complementarily, the non-selective COX inhibitors ketorolac and indomethacin were found to potentiate the expression of NF-κB target genes in brain capillaries and parenchymal microglia during systemic inflammation (i.v. bolus of LPS, IL-1β, or TNF-α) by hampering the activation of the HPA axis and the subsequent release of GCs. Interestingly, this effect could be replicated by the administration of the GR antagonist RU486 (mifepristone) (135, 136).

Glucocorticoids also exert salient anti-inflammatory actions when the immunogenic insult is taking place within the CNS compartment. Accordingly, Nadeau et al. reported that the robust inflammatory response induced by an intracerebral injection of LPS could be abolished by a prior systemic administration of the endotoxin (137). Further investigation revealed that the immunosuppressive actions of peripheral LPS were mediated by plasma corticosterone and GR activation. Indeed, exogenous GCs mimicked whereas the GR antagonist RU486 nullified the anti-inflammatory effect of the systemic immune challenge. The physiological importance of the GC feedback in controlling innate immune responses in organs as sensitive as the brain was further illustrated in rodents and mice injected with RU486 before intracerebral LPS. Antagonizing the GR significantly exacerbated and prolonged the inflammatory response, which in turn provoked substantial neuronal death and even mortality (138, 139). Interestingly, these findings were replicated in GR^lysMCre^ (vs. WT) mice challenged with an intra-striatal bolus of LPS (116), again supporting a pivotal of GR signaling in microglia in restraining the amplitude and duration of the immune response in this context.

### Pro-inflammatory actions of GCs in the brain

The vast majority of GC pro-inflammatory actions *in vivo* were described in animal models of acute or chronic stress. As a matter of act, both types of stress elicit an HPA response and were shown to exacerbate salient features of inflammation in the CNS provided that they occur prior to peripheral or cerebral immune insults. For instance, acute stressors such as inescapable tailshocks (IS) were demonstrated to potentiate the expression of pro-inflammatory mediators in specific regions of the brain following the peripheral administration of LPS in rodents (140–142). Chronically stressed rodents exhibited enhanced NF-κB activity in multiple limbic regions during LPS-induced systemic inflammation (143). Chronically stressed animals injected either in the prefrontal cortex (144) or the hippocampus (145) with this endotoxin also displayed exacerbated microglia activation and tissue damage. Interestingly, in these studies the priming effects of stress (regardless of its chronicity) on the ensuing immune response could be abated with RU486 or by experimentally maintaining plasma GCs to basal levels in adrenalectomized animals. GR signaling was thus proposed to be essential for the cross-sensitization between stress and the inflammatory response to LPS. More specifically, exposure to high levels of GCs was suggested to prime the reactivity of microglial cells to a subsequent immune stimulation presumably by making the neuroimmune environment more permissive to inflammation, inducing GC resistance or blunting the HPA response (146). In support of this hypothesis, restraint stress and other physical stressors were shown to induce inflammatory mediators (e.g., iNOS, TNF-α, COX-2, PGE_2_, IL-1β, and CD14), reduce immunoregulatory proteins (e.g., CD200R) and trigger microglia proliferation in the rodent brain (147–152). In opposition, microglia GR signaling was recently found to suppress rather than bolster the priming effects of chronic stress on microglia reactivity to a subsequent intracerebral injection of LPS (116). Further investigation is needed to shed more light on the factors entailing these conflicting experimental observations about the specific role of microglial GR signaling the priming effect of stress.

Interestingly, GR ligation by endogenous GCs was also found to be required for the expression of several inflammatory genes. Notably, the pharmacological ablation of GR signaling with RU486 was found to hinder the LPS-mediated expression of various immune genes involved in host defense, such as IL-1β (153). Recent data also indicate that the concomitant signaling of GCs and damage-associated molecular patterns (DAMPs) accentuate the expression of pro-inflammatory mediators. In fact, the combined exposure of human and murine myeloid cells to dexamethasone and ATP following LPS was found to enhance the production of IL-1β, TNF-α, IL-6, and IL-10 (43). Synergistic effects of dexamethasone and ATP in the expression of the purinergic receptor P2Y_2_ and key adhesion molecules (e.g., VCAM and ICAM) were also reported (154). Together, these data suggest that GCs can exert pro-inflammatory actions in the context of acute cellular damage or death. Complementarily, dexamethasone and TNF-α were recently shown to coregulate a unique set of immune genes when combined (155). Therefore, as for the anti-inflammatory actions of GCs, the activation state and signaling context of a target cell also define its pro-inflammatory functions.

### Parameters determining the dichotomous roles of GCs in the CNS

Numerous parameters were found to influence the dichotomous actions of GCs. Of particular interest is the timing of GC exposure relative to an immune challenge. Prior work established that acute stress or exogenous GCs can potentiate or conversely repress the same pro-inflammatory genes provided that they are respectively administered before or after LPS (140, 156). In another study, both physical and psychogenic stressors could abate LPS-induced neuroinflammation, as long as they occurred after the immune challenge (157). Timing of exposure to GCs following an immunogenic challenge thus appears to be a pivotal parameter dictating the opposite actions of the steroid hormone (158). The disparate susceptibility of distinct brain regions to innate immune events and GC signaling also represent decisive factors. For example, the prefrontal cortex and hippocampus were both found to be more sensitive than the hypothalamus to inflammation and the neurotoxic effects of GCs (142, 143, 159). Finally, other elements such as the length of exposure, dose, and route of administration as well as the type of synthetic GCs employed should be taken into account (160). In that regard, we noted that pharmacological compounds targeting the GR (agonists and antagonists) are invariably administered via peripheral routes *in vivo* and are rarely quantified in the CNS despite the distribution impediment caused by the BBB. Consequently, one must not exclude that significant immune and/or metabolic disturbances in the periphery may concomitantly influence the immune state of the brain. We also emphasize on the fact that pro-inflammatory markers such as the cytokines such as IL-1β and TNF-α also act in a context-dependent manner. Such cytokines can thus be harmful for the cerebral elements (138) but they also play beneficial roles in the inflammatory response (161–163). It should also be kept in mind that a pro-inflammatory environment does not automatically lead to collateral damages and that timing is again a key parameter here. While an exaggerated (magnitude or duration) inflammatory response can trigger neuronal injury and cell death, pro-inflammatory mediators may initially (i.e., acute phase) stimulate the clearance of debris, the recruitment of cellular reinforcements and program tissue remodeling. Despite complicating the overall picture, such factors must be carefully considered when interpreting the actions of GCs in the CNS.

## Conclusion

A wealth of experimental and clinical data provides clear evidence that accurate signaling between the nervous, endocrine, and immune systems leading to a proper feedback (timing, amplitude, duration, sensitivity, etc.) by GCs is mandatory to avoid serious detrimental consequences for the brain elements following an immunogenic challenge. GCs induce important plastic changes in the brain and many of their effects, including those related to their priming and pro-inflammatory properties may play critical roles in the “yin and yang” effects of the innate immune reaction in the brain. In future studies, it will be critical to decipher the genomic and non-genomic functions underlying the contextual remodeling of the cerebral innate immune system by endogenous and synthetic GCs. Such research endeavor may unravel why GCs either succeed or fail in managing specific inflammatory and neuropathological diseases. Novel regulatory mechanisms of the HPA axis and GC/GR functions, such as those involving epigenetics or miRNAs, also represent promising avenues. Shedding more light on these intricacies will help to better dissect the beneficial and detrimental actions of GCs in the CNS and to develop new therapeutic strategies enabling to balance their paradoxical actions.

## Conflict of Interest Statement

The authors declare that the research was conducted in the absence of any commercial or financial relationships that could be construed as a potential conflict of interest.
